# APPLES TO APPLES? DISCORDANT DEFINITIONS STILL HINDER EVIDENCE-BASED TREATMENTS FOR SARCOPENIA

**DOI:** 10.1093/geroni/igae098.1958

**Published:** 2024-12-31

**Authors:** Giulia Coletta

**Affiliations:** McMaster University, Hamilton, Ontario, Canada

## Abstract

The definition of sarcopenia continues to evolve, creating difficulty in determining the diagnosis and prognosis of the newly classified disease. The most common definitions include a combination of (a) muscle mass [measured using proxies of muscle mass—appendicular lean soft tissue via dual-energy X-ray absorptiometry (DXA) or bioelectrical impedance analysis (BIA)]; (b) muscle strength (often measured using hand grip strength); and (c) physical function (measured using gait speed). However, each consensus definition uses different combinations of muscle mass, strength and physical function to operationalize the definition of sarcopenia. Additionally, each group recommends various measures and cutoff points to capture these outcomes. For example, the European Working Group on Sarcopenia in Older People (EWGSOP) recommends appendicular lean mass for muscle mass, grip strength or chair stand for muscle strength, and gait speed, short performance physical battery, timed up and go, or 400-m walk test for physical function. The Asian Working Group for Sarcopenia (AWGS) uses DXA or BIA, grip strength and 6-m gait speed for muscle, strength and function, respectively. Using different consensus group definitions results in differences in the prevalence of sarcopenia, and there would be, at best, a modest agreement between the various definitions, which would be attributed to the lack of criterion standards.

